# Supplementary figures and images for: Highly Branched Neo-Fructans (Agavins) Attenuate Metabolic Endotoxemia and Low-Grade Inflammation in Association with Gut Microbiota Modulation on High-Fat Diet-Fed Mice
Source: Foods. 2020 Dec 3;9(12):1792. doi: 10.3390/foods9121792 (PMC7761524; doi:10.3390/foods9121792)

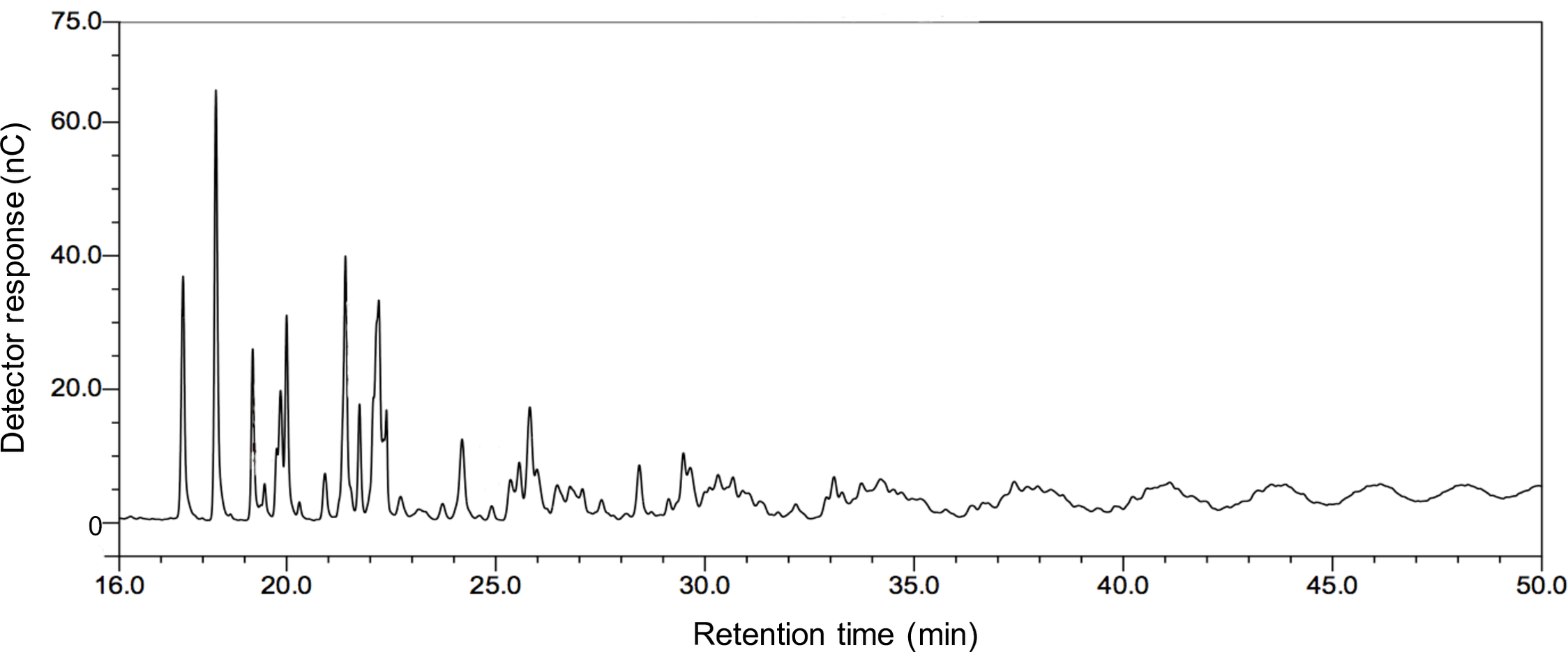

Supplement: Supplementary file 1 [file foods-09-01792-s001.zip › FigureS1.tif]

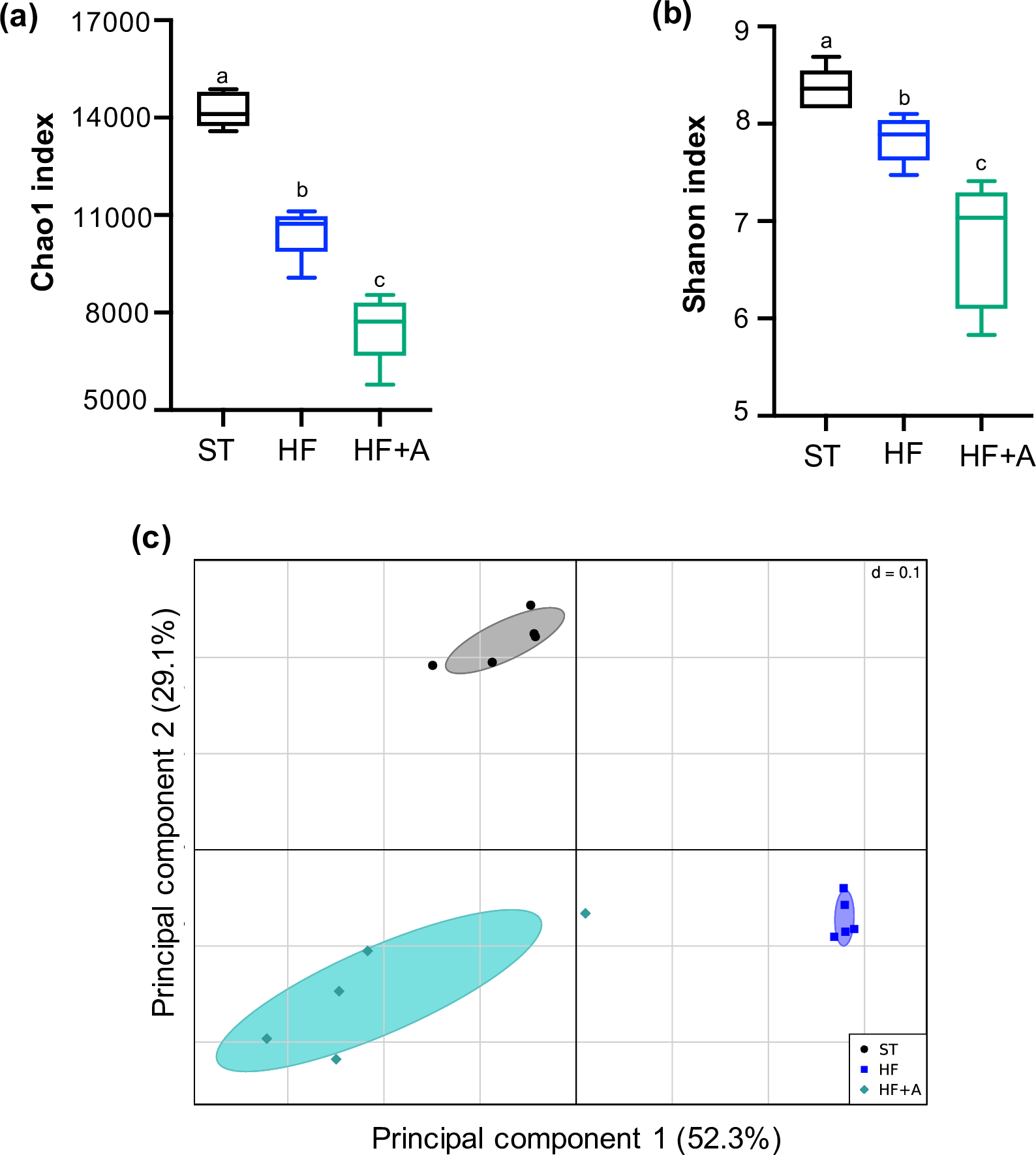

Supplement: Supplementary file 1 [file foods-09-01792-s001.zip › FigureS2.tif]

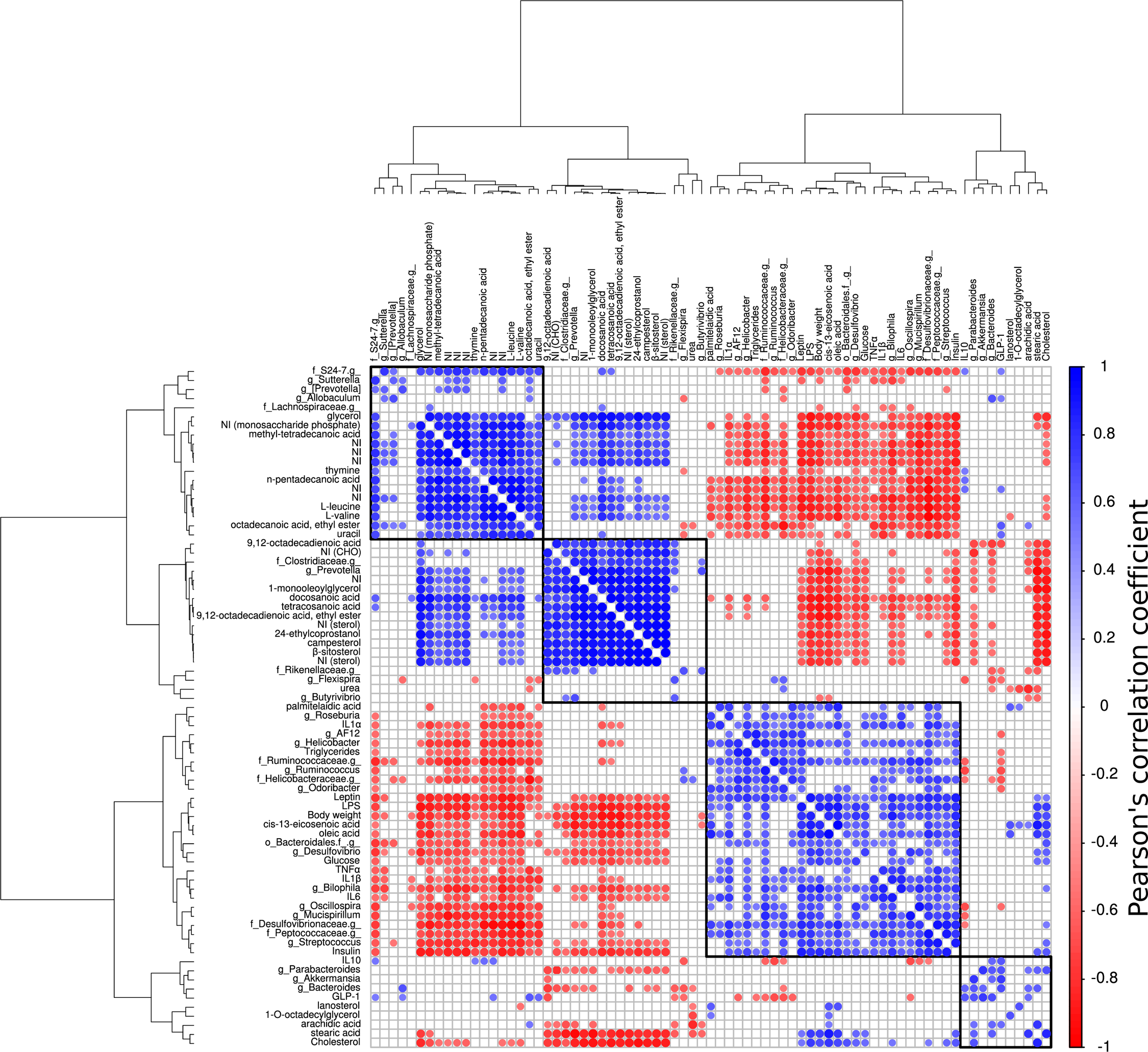

Supplement: Supplementary file 1 [file foods-09-01792-s001.zip › FigureS3.tif]
